# EEG Microstate Sequences From Different Clustering Algorithms Are Information-Theoretically Invariant

**DOI:** 10.3389/fncom.2018.00070

**Published:** 2018-08-27

**Authors:** Frederic von Wegner, Paul Knaut, Helmut Laufs

**Affiliations:** ^1^Epilepsy Center Rhein-Main, Goethe University Frankfurt, Frankfurt, Germany; ^2^Department of Neurology and Brain Imaging Center, Goethe University Frankfurt, Frankfurt, Germany; ^3^Department of Neurology, University Hospital Kiel, Kiel, Germany

**Keywords:** EEG microstates, information theory, entropy, mutual information, markovianity, stationarity

## Abstract

We analyse statistical and information-theoretical properties of EEG microstate sequences, as seen through the lens of five different clustering algorithms. Microstate sequences are computed for *n* = 20 resting state EEG recordings during wakeful rest. The input for all clustering algorithms is the set of EEG topographic maps obtained at local maxima of the spatial variance. This data set is processed by two classical microstate clustering algorithms (1) atomize and agglomerate hierarchical clustering (AAHC) and (2) a modified K-means algorithm, as well as by (3) K-medoids, (4) principal component analysis (PCA) and (5) fast independent component analysis (Fast-ICA). Using this technique, EEG topographies can be substituted with microstate labels by competitive fitting based on spatial correlation, resulting in a symbolic, non-metric time series, the microstate sequence. Microstate topographies and symbolic time series are further analyzed statistically, including static and dynamic properties. Static properties, which do not contain information about temporal dependencies of the microstate sequence include the maximum similarity of microstate maps within and between the tested clustering algorithms, the global explained variance and the Shannon entropy of the microstate sequences. Dynamic properties are sensitive to temporal correlations between the symbols and include the mixing time of the microstate transition matrix, the entropy rate of the microstate sequences and the location of the first local maximum of the autoinformation function. We also test the Markov property of microstate sequences, the time stationarity of the transition matrix and detect periodicities by means of time-lagged mutual information. Finally, possible long-range correlations of microstate sequences are assessed via Hurst exponent estimation. We find that while static properties partially reflect properties of the clustering algorithms, information-theoretical quantities are largely invariant with respect to the clustering method used. As each clustering algorithm has its own profile of computational speed, ease of implementation, determinism vs. stochasticity and theoretical underpinnings, our results convey a positive message concerning the free choice of method and the comparability of results obtained from different algorithms. The invariance of these quantities implies that the tested properties are algorithm-independent, inherent features of resting state EEG derived microstate sequences.

## 1. Introduction and background

Electroencephalography (EEG) records the brain electrical potential from the scalp. In a task-free or resting state condition, the electrical potential shows temporal oscillations in the frequency range of approximately 1–70 Hz. In terms of amplitude, these oscillations are dominated by the so-called alpha rhythm, an amplitude-modulated 8–12 Hz rhythm that characterizes the wakeful rest condition (Niedermeyer and da Silva, 2005). In the spatial domain, the cortical potential also displays characteristic patterns varying over time. EEG microstate analysis aims to characterize these patterns using data compression or clustering techniques. The clustering algorithm reduces the complete set of spatial patterns, provided as input data, to a small, representative set of EEG topographic maps. Thanks to advances in the fields of data science and machine learning in recent years, a large number of clustering algorithms now exist in the literature. These algorithms mainly differ in how they define cluster membership and in their definition of cost functionals to be optimized (Xu and Tian, 2015). For EEG microstate analysis, two methods derived from classical clustering algorithms cover the majority of the existing literature. These methods are the modified K-means algorithm (Pascual-Marqui et al., 1995; Murray et al., 2008) and the atomize and agglomerate hierarchical clustering (AAHC) algorithm (Murray et al., 2008; Brunet et al., 2011). Also, principal component analysis (PCA) and independent component analysis (ICA) have been proposed for microstate research, but are only found in relatively few publications (Skrandies, 1989; Spencer et al., 1999, 2001; De Lucia et al., 2010; Yuan et al., 2012).

The neurobiological relevance of EEG microstates has been studied for physiological and pathological conditions. Among these, the reader can find resting-state experiments (Britz et al., 2010; Musso et al., 2010; Van de Ville et al., 2010; Brodbeck et al., 2012), different task-related conditions (Dimitriadis et al., 2013, 2015; Milz et al., 2015; Dimitriadis and Salis, 2017) and sleep (Brodbeck et al., 2012). Clinical conditions include schizophrenia (Koenig et al., 1999), Alzheimer's disease (Nishida et al., 2013), and narcolepsy (Kuhn et al., 2015; Drissi et al., 2016). An overview of the field has recently been published in two reviews (Khanna et al., 2015; Michel and Koenig, 2017).

Although the variety of clustering algorithms is widely available to researchers, thanks to implementations in basically all programming languages, the interpretation of EEG microstate results obtained with different clustering methods is not obvious. In particular, when comparing studies performed with different clustering algorithms, it is not clear if diverging results should be attributed to the fact that each algorithm extracts different data features or if we are observing differences in the neurobiological processes actually occurring in the brain.

We therefore implemented five clustering methods and applied them to a set of 20 resting state EEG recordings acquired from healthy subjects. We computed a number of statistical and information-theoretical quantities commonly employed to describe microstate data. Our main question concerns the invariance of statistical results across clustering algorithms. In other words, we try to identify robust neurobiological features that do not depend on the chosen algorithm.

As microstates computed by different algorithms can display different geometries, the standard labeling A-D used to label the four canonical microstate maps (Koenig et al., 1999) cannot be applied unequivocally. Even if applied, properties of a certain map from one clustering algorithm may not be comparable with any of the maps produced by another algorithm, in case it has a unique geometry. Therefore, we chose to exclusively use quantities that do not depend on a specific labeling of the microstates. In theoretical terms, all quantities we computed are symmetric with respect to the microstate labeling, i.e., any permutation of microstate labels does not affect the result. Moreover, all quantities used in this work are not restricted to the use of four microstates but can also be calculated for any other number of clusters.

## 2. Materials and methods

### 2.1. Experimental data

A set of EEG recordings from 20 right-handed healthy subjects during wakeful rest (age range: 19–27, mean age: 22,5 years, 7 male) was recorded during a wakeful rest condition. We reported the detailed pre-processing pipeline before (von Wegner et al., 2016, 2017). For each subject, we selected a 120 s segment showing clear posterior alpha oscillations and no movement, eye-blink or electrode artifacts. The 30 channel EEG raw data was sampled at 5 kHz using the standard 10–10 electrode configuration. The pre-processing steps were: (i) band-pass filtering to the 1–30 Hz range using a sixth order zero-phase Butterworth filter with a slope of 24 dB/octave, (ii) down-sampling to 250 Hz, and (iii) re-referencing to an average reference. Written informed consent was obtained from all subjects and the study was approved by the ethics committee of the Goethe University, Frankfurt, Germany.

### 2.2. Microstate analysis

In the following, we will describe the basic properties of the clustering algorithms used and give implementation details to facilitate the reproduction of the results. The code used in this manuscript is contained in our Github repository eeg_microstates.

The input to all clustering algorithms is a set of multi-channel EEG signals. The computational data structure is an array of floating point numbers denoted *X*_*ij*_, with dimensions (*n*_*t*_, *n*_*ch*_), where *n*_*t*_ is the number of temporal samples, and *n*_*ch*_ is the number of EEG channels.

The voltage time series at EEG channel *j* is found as column *j* of the array:

$$\begin{array}{ll} X_{•j} & =X_{ij},\;i=0,\ldots ,n_{t}-1 \end{array}$$

and the voltage topography at time point *i* is stored in the i-th row

$$\begin{array}{l} X_{i•}=X_{ij},\;j=0,\ldots ,n_{ch}-1. \end{array}$$

For average reference data, we automatically have $\underset{j}{\sum }X_{ij}=0$ for all *i*, which facilitates the computation of correlation terms. To simplify notation, we will make consistent use of indices (time *i* = 0, …, *n*_*t*_ − 1, EEG channel *j* = 0, …, *n*_*ch*_ − 1, and microstate label *l* = 0, …, *M* − 1) and therefore omit the explicit limits of sums.

The global field power (GFP) σ_*i*_ at time point *i* is defined as the spatial standard deviation of the instantaneous EEG topography:

$$\begin{array}{ll} \sigma_{i} & =\sqrt{\frac{\sum_{j}X_{ij}^{2}}{n_{ch}-1}}. \end{array}$$

We here follow the approach that only EEG topographies at local GFP maxima are presented to the algorithm, a technique based on the observation that the GFP time series periodically achieves local maxima and that EEG topographies are most clearly defined at these maxima (Lehmann et al., 1987; Strik and Lehmann, 1993; Wackermann et al., 1993; Koenig et al., 1999). Our group followed this approach in all our previous publications on EEG microstates (Brodbeck et al., 2012; Kuhn et al., 2015; von Wegner et al., 2017). To find local maxima of the GFP time series, we first compute the discrete time derivative δ_*i*_ = σ_*i*+1_ − σ_*i*_, and then identify local GFP maxima as the set of time points *I*_max_ where the sign of δ_*i*_ crosses from positive to negative values, i.e.,

$$\begin{array}{ll} I_{\text{max}} & =\left\{i|\text{sgn}\left(\delta_{i}\right)-\text{sgn}\left(\delta_{i-1}\right)=-2\right\}. \end{array}$$

The number of GFP maxima is denoted *n*_max_ = |*I*_max_|. The input data set to all clustering algorithms is denoted $\tilde{X}$, where $\tilde{X}=X_{ij},\;i\in I_{\text{max}}$.

Each clustering algorithm, using one of the methods detailed below, yields a set of *M* microstate maps that represent the EEG data set. Computationally, the microstate maps are stored in an array *A*_*lj*_, with microstate index *l* = 0 … *M* − 1 and channel index *j* = 1 … *n*_*ch*_. In classical microstate analysis (Pascual-Marqui et al., 1995), each EEG topography is represented by exactly one microstate map *A*_*l*•_, rather than using a linear combination of the *M* selected microstate maps. The individual microstate map chosen to represent the EEG topography at time point *i* is determined via a minimum distance, or equivalently, a maximum similarity criterion. The commonly used distance measure between the instantaneous EEG topography *X*_*i*•_ at time point *i*, and the candidate microstate map *A*_*l*•_, is the orthogonal squared distance between both vectors (Pascual-Marqui et al., 1995; Murray et al., 2008):

$$\begin{array}{l} d_{il}^{2}=\underset{j}{\sum }X_{ij}^{2}-{\left(X_{ij}A_{lj}\right)}^{2}. \end{array}$$

Minimizing $d_{il}^{2}$ is equivalent to maximizing the squared covariance between *X*_*i*•_ and *A*_*l*•_:

(1) $$\begin{array}{rc} C_{il}^{2} & ={\left(\underset{j}{\sum }X_{ij}A_{lj}\right)}^{2} \end{array}$$

The computation of microstate sequences is identical for all clustering methods, following a “winner takes all” approach, also called competitive back-fitting (Pascual-Marqui et al., 1995). To achieve this effect, the microstate label *L*_*i*_ at time point *i* is determined by the maximum squared correlation:

(2) $$\begin{array}{rc} L_{i} & =\underset{l}{\text{argmax}}\;C_{il}^{2}. \end{array}$$

To measure how well the microstate sequence approximates the underlying EEG data set, a frequently used quantity is global explained variance (GEV) (Murray et al., 2008). GEV measures the percentage of data variance explained by a given set of microstate maps. The GEV value for a specific microstate map with label *l* is:

(3) $$\begin{array}{rc} \text{GE}{\text{V}}_{l} & =\frac{\sum_{i}\sigma_{i}^{2}C_{il}^{2}\delta_{l,L_{i}}}{\sum_{i}\sigma_{i}^{2}} \end{array}$$

where δ_*l*,*L*_*i*__ is the Kronecker delta, i.e., δ_*l*,*L*_*i*__ = 1 for *L*_*i*_ = *l*, and δ_*l*,*L*_*i*__ = 0 otherwise. The total global explained variance (GEV) is the sum of the GEV values over all microstate maps

(4) $$\begin{array}{rc} \text{GEV} & =\underset{l}{\sum }\text{ GE}{\text{V}}_{l}. \end{array}$$

To further facilitate information-theoretical computations, the microstate labels are stored directly as array indices (0, …, *M*−1) that can be used to compute the discrete distributions that appear in information-theoretical functionals.

#### 2.2.1. Number of clusters

All clustering algorithms used here can be run to yield different numbers of microstate maps and the optimum number can be defined in numerous ways. To keep our analysis compact, we chose to use a fixed number of four microstates for all algorithms. This is useful with respect to the subsequent information-theoretical analyses, but also represents a clear limitation with regard to the optimum performance of the different clustering algorithms. In particular, when focusing on the compressing and representative power of a specific clustering algorithm, the optimum number of clusters (microstate maps) should be computed based on one of several optimization criteria (Murray et al., 2008). The focus of the present work, however, is the comparison of several information-theoretical quantities based on entropy-like expressions. These absolute values of these quantities depend on the size of the discrete distributions and thus on the number of microstate maps. As a simple example, the maximum Shannon entropy of any sequence of *M* labels (microstate maps) is *H*_max_ = log(*M*). The situation becomes more complicated for other functionals, that all depend on *M*. To summarize, the presented analyses allow for direct comparison between the numerical values of the quantities introduced in the following. Future developments may use a combined approach testing optimum cluster numbers for each algorithm, and compare information-theoretical quantities computed for different *M*, simultaneously. However, the reader should note that all methods used in this work and the referenced source code work for any number of microstates *M* > 1.

### 2.3. Atomize and agglomerate hierarchical clustering (AAHC)

The AAHC algorithm as described in Murray et al. (2008) is a deterministic hierarchical clustering method. Determinism means that given a specific input data set, the algorithm will always follow the same steps and yield the same microstate maps for repetitive runs of the algorithm. The AAHC algorithm uses a bottom-up approach in the sense that the algorithm is initialized with a large number of clusters, and then reduces the number of clusters by one during each iteration step. As for all other clustering algorithm implementations used here, AAHC is presented with the EEG topographies at local GFP maxima ${\tilde{X}}_{i•},\;i\in I_{\text{max}}$ (Lehmann et al., 1987; Wackermann et al., 1993; Koenig et al., 1999). For the bottom-up AAHC approach, reducing the input data size is crucial to achieve feasible computation times. For instance, a 200 s. EEG recording down-sampled to 250 Hz yields 50,000 input vectors, in case all vectors are used for initialization. Using GFP maxima only, and assuming a dominant alpha rhythm of approximately 10 Hz, with two GFP maxima per alpha cycle, the input data set can be reduced to 4,000 EEG data vectors. In the initial assignment, each of these topographies is chosen to represent a cluster:

$$\begin{array}{ll} A_{i•} & ={\tilde{X}}_{i•},\;i\in I_{\text{max}}. \end{array}$$

Thus, the initial clusters contain a single data vector ${\tilde{X}}_{i•}$. At each iteration, the worst cluster is disintegrated (atomized) and its members are re-assigned to the remaining clusters, thereby reducing the number of clusters by one (Murray et al., 2008; Brunet et al., 2011; Khanna et al., 2014). To this end, the microstate sequence *L*_*i*_ according to the current microstate map assignment is computed using Equations 1 and 2. Next, for each microstate map, the GEV value is computed according to Equation 3. The worst cluster is the one with the lowest GEV value and is indexed by $l_{\text{min}}=\underset{l}{\text{argmin}}\text{GE}{\text{V}}_{l}$. Each member of the worst cluster is re-assigned to one of the remaining clusters *l* ≠ *l*_min_ according to maximum similarity, again using the squared correlation coefficient as in Equation 2. The iteration stops when the desired number of clusters is reached.

The deterministic cluster calculation is a big advantage as a given data set will always lead to the same clustering results, facilitating the reproducibility of results. It is important to note that reproducibility in this context refers to a fixed data set, and does not refer to either test-retest-reliability for different recordings from the same subject (Khanna et al., 2014), or to cross-validation experiments where clustering is performed on a training data set and validated on the remaining test data.

The main drawback of the AAHC algorithm is the relatively low computational speed. Even though we did not perform a formal benchmarking of the algorithm, it is by far the slowest method among those presented. A Python (Numpy) implementation running on a 64-bit Linux OS and a Quad-Core Intel architecture resulted in computation times of several hours for data sets containing approximately 4,000–5,000 GFP maxima. The algorithm should allow for parallelization to improve speed, but to the best of our knowledge, parallel implementations are still unavailable.

### 2.4. Modified K-means clustering

The modified K-means algorithm as detailed in Pascual-Marqui et al. (1995) and Murray et al. (2008) is a stochastic clustering method based on a linear model of the EEG data. Each EEG data vector (topography) *X*_*i*•_ is modeled as the linear combination of *M* representative microstate maps *A*_*l*•_, with microstate index *l* = 0…*M* − 1, and a residual vector ϵ_*i*•_ composed of *n*_*ch*_ identically and independently distributed Gaussian random variables. The general expression for this data model is

$$\begin{array}{l} {\tilde{X}}_{i•}=\underset{l}{\sum }\alpha_{il}A_{l•}+\epsilon_{i•} \end{array}$$

and a solution can be found by minimizing the cost functional (Pascual-Marqui et al., 1995)

$$\begin{array}{l} F=\underset{i}{\sum }\left\|{\tilde{X}}_{i•}-\underset{l}{\sum }\alpha_{il}A_{l•}\right\|. \end{array}$$

Competitive back-fitting, as discussed above, chooses *A*_*L*_*i*_•_, the single microstate map with index *L*_*i*_, to represent the instantaneous EEG topography *X*_*i*•_, rather than using a linear combination of *M* microstate maps with coefficients α_*il*_. In terms of the linear model, competitive back-fitting is equivalent to the choice α_*i*_*L*__*i*__ = 1, and α_*il*_ = 0 for *l* ≠ *L*_*i*_.

The algorithm is initialized with *M* randomly selected input vectors ${\tilde{X}}_{i•},i=0,\ldots ,M-1$, in our case using EEG data vectors at *M* randomly chosen GFP maxima. The modified K-means algorithm finds local minima of the cost functional in an iterative manner, performing two computational steps per iteration. First, at each iteration the microstate sequence *L*_*i*_ according to the current cluster assignment *A*_*l*•_ is calculated using Equations 1 and 2. In the second step, the microstate maps are updated using the microstate label sequence *L*_*i*_ computed in the first step. The updated microstate map *A*_*l*•_ is given as the normalized eigenvector to the largest eigenvalue of the matrix *S*_*l*_ defined as (Pascual-Marqui et al., 1995):

$$\begin{array}{ll} S_{l} & =\underset{i:\;L_{i}=l}{\sum }{\tilde{X}}_{i•}^{\prime }{\tilde{X}}_{i•} \end{array}$$

Using the eigenvector method is equivalent to solving

$$\begin{array}{ll} A_{l•} & =\underset{\;U}{\text{argmax}}{\;U}^{\prime }S_{l}U \end{array}$$

where the maximum is computed across all *n*_*ch*_-dimensional column vectors *U*, subject to ∥*U*∥ = 1. Knowledge of *L*_*i*_ is necessary to select the correct indices in the above sum. The sequence *L*_*i*_ essentially defines the cluster assignments and changes during the optimization procedure. Convergence is assessed using the relative change in residual variance. Given the microstate assignment *L*_*i*_, the residual variance with respect to EEG data at GFP maxima ($\tilde{X}$) is proportional to Pascual-Marqui et al. (1995):

(5) $$\begin{array}{rc} \sigma_{L}^{2} & \propto \underset{i}{\sum }\underset{j}{\sum }{\tilde{X}}_{ij}^{2}-{\left({\tilde{X}}_{ij}A_{L_{i}j}\right)}^{2}. \end{array}$$

The normalization constant to obtain the correct variance value can be omitted for optimization purposes, as it does not change over iterations. In terms of EEG topographies, the eigenvector method implicitly ignores the polarity of the maps as the eigenvector simply defines a direction in the *n*_*ch*_-dimensional EEG sensor space. As the initialization step is stochastic, the resulting microstate maps differ for different runs of the algorithm. To approach the global maximum of the optimization problem, it is recommended to run the K-means algorithm various times. We chose the best out of ten runs to define microstates. For each run of the algorithm, we set the convergence criteria to a maximum number of 500 iterations and a relative error in $\sigma_{L}^{2}$ of 10^−6^. The best run of the algorithm is chosen according to the cross-validation criterion CV given in Murray et al. (2008):

$$\begin{array}{ll} \text{CV} & ={\hat{\sigma }}_{L}^{2}{\left(\frac{n_{ch}-1}{n_{ch}-1-M}\right)}^{2} \end{array}$$

where ${\hat{\sigma }}_{L}^{2}$ is calculated with Equation 5, but including the time indices *i* = 0, …, *n*_*t*_ − 1 of the entire EEG data set *X*_*ij*_. The best run of the modified K-means algorithm is the one with the minimum CV value.

K-means is a fast, commonly used method for which pseudo-code can be found in the original article (Pascual-Marqui et al., 1995). A detailed introduction to our open-source implementation (eeg_microstates) is given in von Wegner and Laufs (2018), where further Matlab and Python implementations by other groups are also listed.

### 2.5. K-medoids clustering

K-medoids is a method highly similar to K-means but calculates cluster centroids, i.e., microstate maps by a median operator, rather than by the arithmetic mean or the maximum eigenvector of the cluster members (Park and Jun, 2009; Xu and Tian, 2015). This means that each cluster representative is an actual input data vector. In our context, each microstate map produced by this procedure represents an actual EEG topography as recorded during the experiment. In contrast, the AAHC/K-means microstate maps correspond to averaged topographies or eigenvectors, respectively. Thus, the microstates obtained from the AAHC/K-means algorithms theoretically may lack neurobiological significance and could actually represent biophysically impossible potential distributions. On the other hand, K-medoids focuses on a specific map to represent the cluster, and the lack of averaging may lead to a sub-optimum representation of other important topographies. In analogy to the modified K-means algorithm, we ran the algorithm ten times and allowed for a maximum of 500 iterations. Similar to K-means, K-medoids is a stochastic algorithm with short computation times. Matlab code for the K-medoids algorithm can be found in several open-source projects (e.g., kmedoids1 and kmedoids2), as well as in the commercial Matlab statistics and machine learning toolbox. Our Python implementation is part of our Github repository.

### 2.6. Principal component analysis (PCA)

Principal component analysis (PCA) is one of the most frequently encountered clustering algorithms with a straightforward statistical interpretation (Haykin, 1999). Spatial PCA has been used to cluster EEG topographies mainly in the context of ERP experiments (Skrandies, 1989; Spencer et al., 1999, 2001). Clusters, or principal components, correspond to eigenvectors of the data covariance matrix

$$\begin{array}{ll} Q & =\frac{1}{n_{\text{max}}-1}{\tilde{X}}^{\prime }\tilde{X} \end{array}$$

where $\tilde{X}$ contains the EEG data vectors at GFP peaks, and the prime denotes matrix transposition. *Q* is a symmetric square matrix of size *n*_*ch*_. Due to symmetry, the eigenvalues are all real-valued and the eigenvectors are mutually orthogonal. The matrix *Q* can be reconstructed exactly from the complete set of eigenvalues λ_*i*_ and the row eigenvectors *a*_*i*•_, for *i* = 0, …, *n*_*ch*_ − 1:

(6) $$\begin{array}{rc} Q & =\underset{i}{\sum }\lambda_{i}a_{i•}^{\prime }a_{i•}. \end{array}$$

The PCA algorithm produces a partition of the total data variance, such that the sum of the single component variances is equal to the total variance of the data set. Moreover, the PCA algorithm yields a natural ordering of components in terms of eigenvalues and explained variance, as the variance contribution of each principal component decreases with the norm of the eigenvalue. A common procedure in data compression is to truncate the eigenvector decomposition in Equation 6, using only *M* components. Geometrically, the truncation represents a projection of the multidimensional data set onto a linear subspace of dimension *M*. The truncation can be performed in different ways (Haykin, 1999). One option is to preset a desired threshold of explained variance and then find the dependent variable *M*, such that the threshold criterion is met. Alternatively, the number of components can be fixed at *M*, which renders the amount of explained variance the dependent variable. To be consistent with the other clustering algorithms, we follow the second approach, using *M* = 4. The EEG data set can thereby be reduced from *n*_*ch*_ = 30 to *M* = 4 dimensions. Using the classical microstate approach, we further reduce the data set to a symbolic sequence via competitive back-fitting (Equations 1, 2). This means that only one principal component, or microstate map, is used to represent the EEG data vector *X*_*i*•_.

PCA-based clustering is a deterministic algorithm and thereby useful when reproducibility of the results is a primary target. As argued in the AAHC section, reproducibility refers to a given data set, not to repetitive measurements of a subject. The clustering is efficient in the sense that the clusters are uncorrelated and form orthogonal subspaces of the complete data space. On the other hand, it may be argued that the mandatory orthogonality of clusters may produce microstates that lack biological relevance as the microstate geometry has to comply with the restrictions given by the algorithm, rather than adapting to some biological boundary condition.

PCA is a generic method that facilitates the integration of other electrophysiological or imaging modalities (e.g., magnetencephalography, functional MR imaging) into the same analysis framework. The algorithm is very fast and implementations can be found for the majority of programming languages and in numerous statistical software packages. We used the PCA algorithm contained in the Python scikit-learn statistics and machine learning package.

### 2.7. Fast independent component analysis (Fast-ICA)

Independent component analysis (ICA) is another widely used technique to analyze neurophysiological data sets, especially in the areas of EEG and fMRI, among others (Makeig et al., 1995; Haykin, 1999; Yuan et al., 2012). In the context of EEG microstates, ICA decomposition is not frequently encountered (De Lucia et al., 2010; Yuan et al., 2012). While PCA clustering is based on statistical decorrelation, ICA looks for statistical independence between components (or clusters). In the context of EEG microstates, ICA theory translates to a model in which the EEG topographies are a linear mixture of a set of unknown source topographies. The key construct is the unmixing matrix, denoted *W* in standard ICA notation. The matrix *W* inverts the mixing process, under the additional constraint that the reconstructed source signals are statistically independent. The ICA algorithm intrinsically ignores the signal's polarity. Unmixing can be written in matrix notation as $S=W{\tilde{X}}^{\prime }$, where ${\tilde{X}}^{\prime }$ are the EEG data vectors at GFP maxima and *S* is the so-called source matrix. The unmixing matrix *W* contains the independent components as row vectors. Algorithmic details have been presented numerous times in the literature (e.g., Makeig et al., 1995; Haykin, 1999) and are not repeated here. The algorithm can be setup to yield any number of *M* ≤ *n*_*ch*_ independent components, each of which is interpreted as a microstate map *A*_*l*•_ = *W*_*l*•_ in our setting. We could have used the symbol *A* instead of *W* directly, however this would conflict with standard ICA notation where *A* is the mixing matrix. Readers familiar with ICA literature should be aware of the different use of the symbol *A* in this manuscript. Although anatomical and functional brain networks are strongly connected and therefore often produce statistically dependent signals, the search for independent components may reveal complementary information about brain function and has its own tradition in modern neuroscience. Similar to our arguments regarding the use of PCA, the necessary condition of independent clusters may interfere with biological reality, i.e., functionally important EEG topographies may remain hidden to ICA if they are statistically dependent. A similar argument against ICA to analyze microstates is found in Brunet et al. (2011). However, as the ICA principle is not reflected by the other algorithms, we decided to include ICA in our analysis. We use the Fast-ICA algorithm in its parallel form, with pre-whitening and an exponential activation function, as implemented in the scikit-learn package.

### 2.8. Information-theoretical analysis

In contrast to a metric time series consisting of integer or real numbers, microstate sequences are symbolic time series to which many metric methods (e.g., power spectrum, autocorrelation function) cannot be applied directly. We therefore introduced an information-theoretical approach that uses the microstate labels as random variables directly. The details of this approach have been given in von Wegner et al. (2017) and have been made available as an open-source Python package (von Wegner and Laufs, 2018). We here briefly summarize the methods used in this paper.

The number of times a specific microstate label (A-D) occurs in a microstate sequence gives the empirical distribution of microstate labels *p* = (*p*_*A*_, *p*_*B*_, *p*_*C*_, *p*_*D*_). The shape of this distribution can be characterized by its **Shannon entropy** (Kullback, 1959)

(7) $$\begin{array}{rc} h & =-\underset{i}{\sum }p_{i}\log p_{i} \end{array}$$

where the sum index *i* runs over the set of microstate labels. The minimum value for entropy is *h* = 0 in case of a distribution that has a value *p*_*i*_ = 1 for a specific label *i*, and *p*_*j*_ = 0 for all other labels *j* ≠ *i*. The maximum entropy of any sequence of four symbols is log4, corresponding to a uniform distribution of symbols (*p*_*i*_ = 0.25 for all *i*).

The **joint entropy** *h*_*n*_ for n-dimensional distributions *p*(*x*_1_, …, *x*_*n*_) is given by Kullback (1959)

(8) $$\begin{array}{rc} h_{n} & =-\underset{x_{1},\ldots ,x_{n}}{\sum }p\left(x_{1},\ldots ,x_{n}\right)\log p\left(x_{1},\ldots ,x_{n}\right). \end{array}$$

The **entropy rate** $h_{n}^{\prime }$ of a stochastic process quantifies how much uncertainty or randomness the process produces at each new time step, given knowledge about the past states of the process (Levin et al., 2006):

(9) $$\begin{array}{rc} h_{n}^{\prime } & =-\frac{1}{n}\underset{x_{1},\ldots ,x_{n}}{\sum }p\left(x_{1},\ldots ,x_{n}\right)\log p\left(x_{1},\ldots ,x_{n}\right) \end{array}$$

(10) $$\begin{array}{r} =\frac{1}{n}h_{n} \end{array}$$

where *p*(*x*_1_, …, *x*_*n*_) denotes the joint probability of a specific sequence of microstate labels (*x*_1_, …, *x*_*n*_). In theoretical analyses of stochastic processes with analytically known properties, the entropy rate is calculated as the limit of $h_{n}^{\prime }$ for *n* → ∞. For empirical and finite data sets, as EEG microstate sequences, the entropy rate however has to be estimated from finite dimensional joint distributions, Equation 8 (Lizier et al., 2012). The empirical finite dimensional distributions *p*(*x*_1_, …, *x*_*n*_), and the associated joint entropies *h*_*n*_ are computed using their maximum likelihood estimates (Marton and Shields, 1994), also termed the plug-in estimates in e.g., Lizier (2014).

To approximate Equation 9, the entropy rate $h_{n}^{\prime }$ is estimated as the slope of the linear least squares fit of *n* vs. *h*_*n*_ (Lizier et al., 2012). As the quality of joint entropy estimates quickly deteriorates with increasing *n*, we numerically determined an optimum parameter $\hat{n}$. To this end, we synthesized a first-order Markov surrogate sequence for each EEG microstate sequence, using the microstate sequences' symbol distribution π and transition matrix *T*, as described theoretically in Häggström (2002), and as explained for microstate sequences in von Wegner and Laufs (2018). It is important to use surrogates of the same length as the original data, as the number of samples clearly influences the estimate. The theoretical entropy rate $h_{\text{MC}}^{\prime }$ for the Markov chain surrogate with stationary distribution π and transition matrix *T* is given by $h_{\text{MC}}^{\prime }=-\underset{i}{\sum }\pi_{i}\underset{j}{\sum }T_{ij}\log T_{ij}$ (Levin et al., 2006). We averaged the relative error $\epsilon =\frac{\left|h_{n}^{\prime }-h_{\text{MC}}^{\prime }\right|}{h_{\text{MC}}^{\prime }}$ of our estimate over all subjects for *n* = 4…10, and found that $\hat{n}=8$ was the largest dimension such that ϵ < 0.05. This was found for all clustering algorithms. Therefore, in the following, all entropy rates correspond to estimates $h_{n=8}^{\prime }$.

The expressions for the Markovianity and stationarity tests were derived in Billingsley (1961), Kullback et al. (1962), and applied to characterize microstate sequences in von Wegner et al. (2017), von Wegner and Laufs (2018). Stationarity is assessed for data segments of length *L* = 500 (2 s), *L* = 1, 000 (4 s), *L* = 2, 500 (10 s), *L* = 5, 000 (20 s), and *L* = 10, 000 (40 s). Finally, temporal dependencies in microstate sequences are estimated with the information-theoretical analog of the time autocorrelation function, the autoinformation function (AIF) (von Wegner et al., 2017). The AIF of the stochastic process *X*_*t*_ and for time lag *k* is defined by the mutual information between the random variables *X*_*t*_ and *X*_*t*+*k*_:

(11) $$\begin{array}{rc} I\left(k\right) & =H\left(X_{t+k}\right)-H\left(X_{t+k}|X_{t}\right). \end{array}$$

Further theoretical details and the software implementation can be found in von Wegner et al. (2017), von Wegner and Laufs (2018).

### 2.9. Hurst exponents

Hurst exponents quantify the scaling behavior of the multi-scale variance of random walks and other stochastic processes (Veitch and Abry, 1999; Abry et al., 2002). Long-range correlated stationary processes show a linear increase of their scale-dependent variance as a function of scale, when plotted in log-log coordinates. The Hurst exponent measures the slope of the multi-scale variance estimate and quantifies long-range correlations for stationary processes. For a stationary process, a Hurst exponent of *H* > 0.5 indicates long-range correlations whereas *H* = 0.5 indicates a short-range correlated stationary process. With respect to microstate analysis, a method to apply Hurst exponent estimation to symbolic sequences has been reported (Van de Ville et al., 2010). We implemented three different estimates of the Hurst exponent that we have analyzed earlier for K-means clustered microstates (von Wegner et al., 2016). In particular, we use the aggregated variance (AV) method, detrended fluctuation analysis (DFA) and a discrete wavelet transform (DWT) based estimate. Further details are found in von Wegner et al. (2016).

### 2.10. Static and dynamic properties

In the following, we will group six of the aforementioned quantities into two groups to summarize the statistics of microstate sequences. We distinguish static from dynamic properties and define static properties to be quantities that do not contain information about temporal dependencies within the microstate sequences, whereas dynamic properties capture temporal properties of the symbolic sequences.

#### 2.10.1. Static properties

##### 2.10.1.1. Intra-group correlation

To quantify the similarity between microstate maps, we use a correlation-based measure. Given two microstate maps with *n*_*ch*_ EEG channels each, *u* = (*u*_1_, …, *u*_*n*_*ch*__) and *v* = (*v*_1_, …, *v*_*n*_*ch*__), Pearson's correlation coefficient ρ between *u* and *v* is defined as

(12) $$\begin{array}{rc} \rho \left(u,v\right) & =\frac{\sum_{i=0}^{n_{ch}-1}\left(u_{i}-\mu_{u}\right)\left(v_{i}-\mu_{v}\right)}{\sigma_{u}\sigma_{v}} \end{array}$$

where μ_*u,v*_ and σ_*u,v*_ represent the means and standard deviations of the maps *u* and *v*, respectively. Ignoring polarity, we use the absolute value of the correlation coefficient, |ρ(*u, v*)|, to measure similarity between *u* and *v*. We summarize the similarity between microstate maps produced by a specific clustering algorithm by the maximum absolute value of Pearson's correlation coefficient, Equation 12, across all pairs of maps. When comparing the four maps produced by any given algorithm, the diagonal elements (equal to one) and half of the off-diagonal elements can be omitted due to symmetry, such that six values remain.

$$\begin{array}{ll} \rho_{\text{max}} & =\underset{i>0,\;j>i}{\text{max}}|\rho \left(A_{i•},A_{j•}\right)|. \end{array}$$

Another option would have been to measure maximum dissimilarity by the minimum correlation value between pairs of maps.

##### 2.10.1.2. Global explained variance

We used the global explained variance (GEV) as defined for a single microstate map in Equation 3, and for a set of maps in Equation 4, following (Murray et al., 2008). GEV is computed across the whole sequence and measures the amount of EEG data variance captured by the set of microstate maps. As it does not contain about temporal dynamics, it can be considered a static property.

##### 2.10.1.3. Shannon entropy

Shannon entropy as defined in Equation 7 and Kullback (1959), can be considered as an information-theoretical analog of variance, as it measures the width of the probability distribution of microstate labels. It is averaged across the entire microstate sequence and can also be considered a static property.

#### 2.10.2. Dynamic properties

##### 2.10.2.1. Mixing time

Single time step dependencies (*t* → *t* + 1) of a microstate sequence are often summarized by the so-called transition matrix *T*, where each matrix element *T*_*ij*_ represents the conditional probability of observing microstate label *j* at time *t* + 1, given that microstate label *i* occurred at time *t* (Koenig et al., 2002; Brodbeck et al., 2012; von Wegner et al., 2017). To characterize *T* and compare transition matrices from different clustering algorithms, we use the eigenvalues of *T* as these are invariant under permutations of the microstate labels (von Wegner et al., 2016). The set of four eigenvalues is further summarized by a quantity called spectral gap, λ_0_ − λ_1_, where λ_0_ and λ_1_ are the largest and second largest eigenvalue of *T*, respectively. Furthermore, the Perron-Frobenius theorem for stochastic matrices assures λ_0_ = 1. The mixing time τ of the Markov process represented by *T* is defined as

(13) $$\begin{array}{rc} \tau & =\frac{1}{1-\lambda_{1}}. \end{array}$$

##### 2.10.2.2. Entropy rate

The entropy rate, as defined in Equation 9, measures a temporal property of the microstate sequence as it quantifies the average amount of information produced at each time step. It can be regarded as the averaged derivative of Shannon entropy with respect to time. The entropy rate cannot be larger than the Shannon entropy of a given sequence. For a temporally uncorrelated stationary sequence, the entropy rate is equal to the Shannon entropy.

##### 2.10.2.3. Autoinformation peaks

In the following, the dominant periodicity of a microstate sequence is measured by the location of the first peak of the autoinformation function, Equation 11. We consider periodicity, i.e., oscillatory activity, a temporal property and therefore added the first peak parameter to the set of dynamic properties. The AIF always has an absolute maximum at time lag zero, such that the we search for first local maximum for time lags larger than 8 time steps (32 ms). To reduce the influence of noise, we search for the first peak after smoothing the AIF with a moving average filter of size 3. The actual local maximum is calculated from the discrete derivative as explained above for GFP peaks. Visual inspection of all AIF curves and the automatically computed local maximum showed that no peaks were missed.

## 3. Results

All five algorithms were applied to the 20 resting state EEG data sets and all runs of the tested clustering algorithms converged. To quantify the properties of microstate clusterings produced by different algorithms, we analyse a set of static and dynamic properties for both, the sets of microstate maps, and for the symbolic time series of microstate labels (microstate sequences).

### 3.1. Static properties

#### 3.1.1. Microstate maps

**Figure 1:**
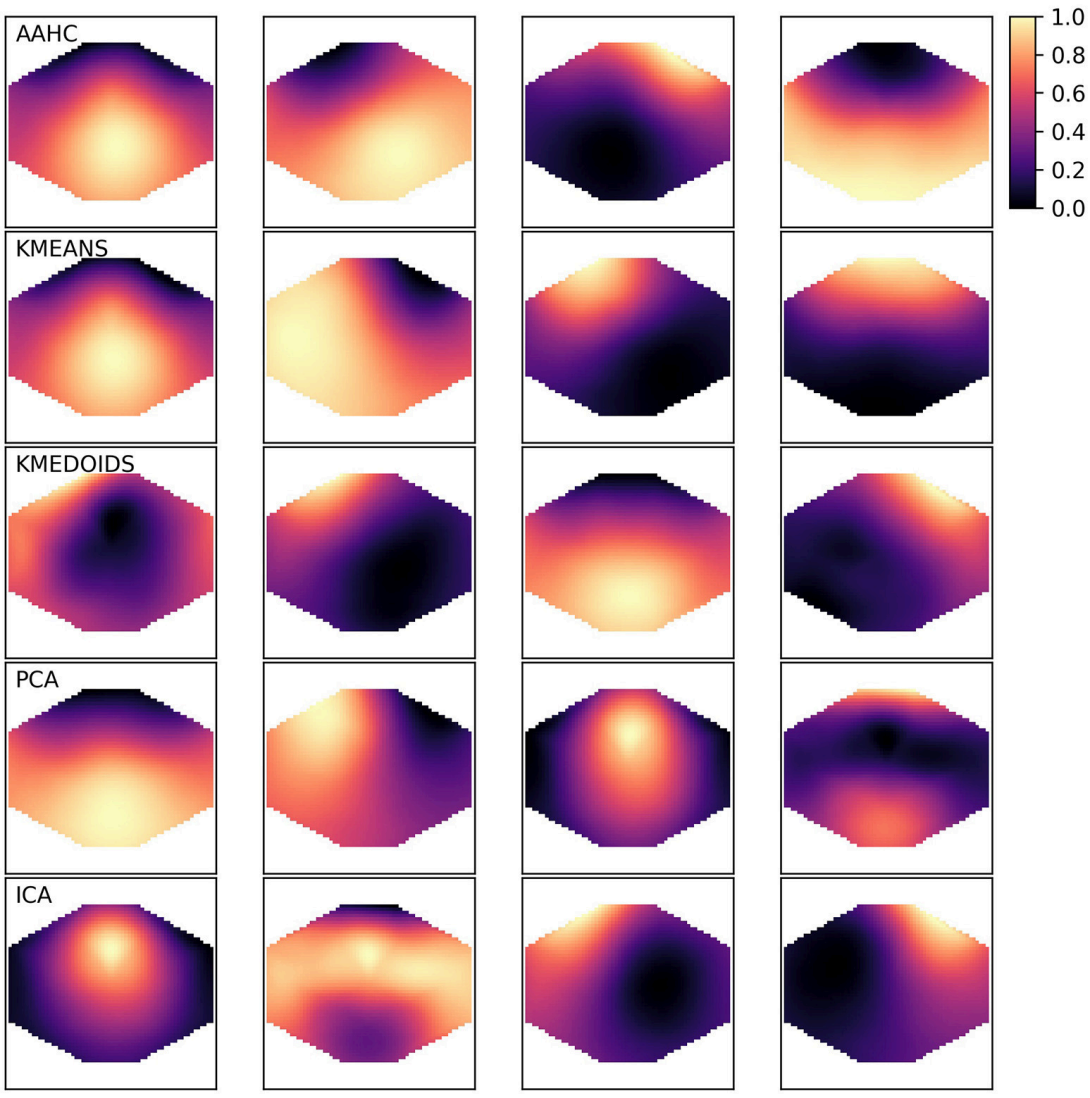
Exemplary microstate maps from a resting state EEG recording of 120 s duration from a single healthy subject. The four microstates produced by each clustering method are shown row-wise and the algorithms are abbreviated in the upper left corner. The color map shown in the upper right corner is valid for all algorithms. As microstate maps are normalized and neither absolute amplitudes nor polarity is used in further analyses, values can be represented in the [0, 1] range.

The canonical microstate map geometries have been described based on two algorithms, AAHC and the modified K-means approach (Lehmann et al., 1987; Pascual-Marqui et al., 1995; Koenig et al., 2002). The resulting maps can be described by their geometry. For microstate A, the border between positive and negative potentials runs approximately along the diagonal from the left frontal to the right occipital right corner of the scalp. For the canonical microstate B, the diagonal runs in the opposite direction. Microstate C has a horizontal orientation and map D is often circular. Sometimes, the classical algorithms (AAHC, K-means) produce different symmetries, mostly one with a vertical border between positive and negative potentials. We observed that the other algorithms presented here produce some new geometries. As these geometries are hard to classify quantitatively and to use in statistics, we will just illustrate a set of microstates for one subject and then proceed with the quantitative analyses. Figure 1 shows five sets of four microstates, one set for each clustering algorithm. We observe the four canonical maps in the first two rows (AAHC, K-means). The canonical microstate labeling for AAHC would be D, B, A, C and the ordering for the K-means results would be D, A, B, C. The K-medoids algorithm shows the canonical maps B, C, A in columns 2–4, but map 1 in the first column is more difficult to classify. Both, PCA and ICA (rows 4 and 5) show a new microstate geometry with a frontal and an occipital maximum (PCA microstate 4, ICA microstate 2), that cannot be mapped unequivocally to one of the canonical microstates. Similarly, the circular maps (PCA microstate 3, ICA microstate 1) are clearly different from the microstates produced by AAHC or K-means.

#### 3.1.2. Inter-group correlations

To quantify the similarity between the microstate maps analyzed in a descriptive way above, using a correlation-based measure. We quantify the similarity between two microstate maps *u* and *v* by the measure defined in Equation 12. As each algorithm yields four microstate maps, the similarities between the maps produced by two clustering algorithms can be summarized in a 4 × 4 correlation matrix *C*_*ij*_ = ρ(*u*_*i*_, *v*_*j*_), where *u*_*i*_ is a map computed by one algorithm, and *v*_*j*_ is a map computed another algorithm. As *C* is symmetric (*C*_*ij*_ = *C*_*ji*_), it contains only ten independent coefficients. To compare two algorithms, we only retain the maximum absolute correlation $c_{\text{max}}=\underset{ij}{\max}|C_{ij}|$ out of each 4 × 4 matrix *C*. To compare the microstate maps of all clustering algorithms, i.e., for each pairwise combination of the five methods, we place the corresponding *c*_max_ value into a 5 × 5 matrix *C*^max^. Each element of *C*^max^ contains the maximum similarity between the microstate maps computed by two different clustering algorithms, the results are given in Table 1. As correlation is symmetric, we omit the values below the diagonal. Likewise, we can omit the diagonal itself as all values are exactly one. The table shows the mean values and their standard errors across the 20 subjects studied. We tested for statistically significant differences between the *C*_max_ values using a one-way ANOVA across the ten possible combinations of clustering algorithms and found *p* = 0.000, i.e., significant differences exist within the similarity matrix. Maximum correlation values range from 0.669 (AAHC vs. ICA) to 0.982 (PCA vs. ICA). The similarity of the two classical algorithms, AAHC and K-means, lies in the mid range (0.825). In total, the microstate maps between any pair of the clustering algorithms show a rather high similarity. As we use maximum correlation, this result does not imply the absence of uncorrelated maps but states that for any pair of algorithms, at least one of the microstate maps shows a high similarity with at least one of the maps produced by the other algorithm.

**Table 1:** Maximum between-group correlations.

**Algorithm**	**AAHC**	**K-means**	**K-medoids**	**PCA**	**ICA**
AAHC	-	0.825 ± 0.086	0.778 ± 0.081	0.776 ± 0.084	0.669 ± 0.103
K-means	-	-	0.962 ± 0.022	0.981 ± 0.015	0.903 ± 0.080
K-medoids	-	-	-	0.949 ± 0.037	0.842 ± 0.091
PCA	-	-	-	-	0.982 ± 0.037
ICA	-	-	-	-	-

*The maximum absolute correlation value between microstate maps from different algorithms and for all subjects. Mean and SD values are given for unique pairs of algorithms*.

#### 3.1.3. Intra-group correlations

Next, we quantify the similarity between the maps found by a specific clustering algorithm, using the maximum absolute value of Pearson's correlation coefficient, Equation 12. When comparing the four maps produced by any given algorithm, the diagonal elements (equal to one) and half of the off-diagonal elements can be omitted due to symmetry, such that six values remain. The results are shown in the first column (ρ_max_) of Table 2. Most notably, the PCA results yield zero correlation between any pair of maps. This fact can be anticipated and indicates correct working of the PCA algorithm as the clusters are represented by the orthogonal eigenvectors of the data covariance matrix. Apart from PCA, ICA shows the lowest intra-group correlations, ρ_max_ ≈ 0.36. The ICA algorithm partially de-correlates the input data but some linear correlation remains between the maps. The other three algorithms yield comparatively large maximum intra-group correlation of approximately 70-80 %. We tested for statistically significant differences between ρ_max_ values using a one-way ANOVA across clustering algorithms and found *p* = 0.000. This result is not surprising as the PCA results, and to a lesser extent also the ICA results are clearly separated from the other algorithms. However, *post-hoc* analysis shows that even when excluding the PCA and ICA results, the maximum correlation values of the other three algorithms are still significantly different.

**Table 2:** Static microstate properties, given as mean ± SD values.

**Algorithm**	**ρ_max_**	**GEV [%/100]**	**Entropy [nats]**
AAHC	0.819 ± 0.061	0.613 ± 0.056	1.360 ± 0.013
K-means	0.734 ± 0.061	0.658 ± 0.049	1.381 ± 0.006
K-medoids	0.890 ± 0.066	0.583 ± 0.063	1.324 ± 0.050
PCA	0.000 ± 0.000	0.611 ± 0.044	1.161 ± 0.062
ICA	0.363 ± 0.131	0.483 ± 0.070	1.233 ± 0.078

#### 3.1.4. Global explained variance

GEV, as defined in Equation 4, measures the percentage of data variance explained by a given set of microstates. Though GEV values can be computed for each of the microstate maps (Equation 3), often the sum over GEV values of all individual microstates is reported. The second column (GEV) of Table 2 shows the results for our data set. We find GEV values ranging from 0.483 to 0.658. The values for AAHC and K-means (0.6−0.7) are in general agreement with the literature (e.g., Koenig et al., 2002; Brodbeck et al., 2012). The GEV for PCA (0.611) is similar to the classical algorithms, whereas K-medoids (0.583) and ICA (0.483) yield rather low values. The one-way ANOVA across all methods shows significant differences between the GEV values of different algorithms (*p* = 0.000). *Post-hoc* exclusion of either ICA or both, PCA and ICA, does not change these findings.

#### 3.1.5. Shannon entropy

While GEV measures how much of the time-varying spatial variance of the EEG signal is captured by the four microstate maps, Shannon entropy as given in Equation 7, measures the amount of information, or uncertainty, contained in the microstate sequence. Shannon entropy is bounded from above by the maximum amount of entropy for any symbolic sequence with four symbols, in our case *h*_max_ = log(4) ≈ 1.386. Shannon entropy measures the shape of the microstate distribution independent of the specific labeling of the maps. The results are shown in the third column of Table 2. A minimum entropy of 1.161 is found for PCA and the maximum value of 1.381 is found for K-means. The one-way ANOVA across all clustering methods, as well as *post-hoc* pairwise comparison of pairs of algorithms shows significant differences between all algorithms.

### 3.2. Dynamic properties

#### 3.2.1. Transition matrix spectra and mixing time

The first column of Table 3 shows the mixing time statistics for the 20 EEG data sets. The ANOVA across methods gives *p* = 0.004, i.e., there are significant difference between the clustering algorithms. *Post-hoc* analysis by means of a two-sided *t*-test for samples with unequal variances shows no significant differences for the comparisons AAHC/K-means (*p* = 0.552), AAHC/K-medoids (*p* = 0.985), AAHC/PCA (*p* = 0.031), AAHC/ICA (*p* = 0.015), K-means/K-medoids (*p* = 0.576), K-medoids/PCA (*p* = 0.033), K-medoids/ICA (*p* = 0.017), and PCA/ICA (*p* = 0.867). Significant differences (*p* < 0.01) are found for the comparisons K-means/PCA (*p* = 0.008) and K-means/ICA (*p* = 0.003).

**Table 3:** Dynamic microstate characteristics, given as mean ± SD values.

**Algorithm**	**Mixing time**	**Entropy rate [nats]**	**AIF-1 [ms]**
AAHC	3.401 ± 0.261	1.094 ± 0.048	49.60 ± 6.62
K-means	3.350 ± 0.261	1.101 ± 0.051	50.00 ± 5.87
K-medoids	3.400 ± 0.275	1.072 ± 0.062	49.80 ± 6.48
PCA	3.602 ± 0.290	0.915 ± 0.056	49.80 ± 5.86
ICA	3.618 ± 0.261	0.960 ± 0.065	50.91 ± 6.38

*Legend*.

#### 3.2.2. Entropy rate

The entropy rate of a stochastic process quantifies how much information or uncertainty is produced by the sequence at each time step. The second column of Table 3 summarizes the entropy rate statistics. ANOVA across methods gives *p* = 0.000, i.e., significant differences between the algorithms. *Post-hoc* analysis with a *t*-test does not show differences between AAHC/K-means (*p* = 0.681), AAHC/K-medoids (*p* = 0.226), K-means/K-medoids (*p* = 0.126), and PCA/ICA (*p* = 0.028).

#### 3.2.3. Periodicity-first AIF peak

We here report the location of the first local AIF maximum as detailed in the Methods section. The value characterizes the dominant periodic component of the underlying microstate sequence. The characteristic frequencies observed are closely related to the dominant EEG frequency band. In particular, we observed that microstate sequences display a dominant wavelength of approximately 50 ms, corresponding to twice the alpha frequency. The results are presented in the third column of Table 3. All algorithms find values around 50 ms, as expected in the case of a dominant alpha frequency of approximately 10 Hz, i.e., an oscillation with period 100 ms. Using a one-way ANOVA across methods, we do not find significant differences between the first-peak locations of the autoinformation functions computed for different clustering algorithms.

**Figure 2:**
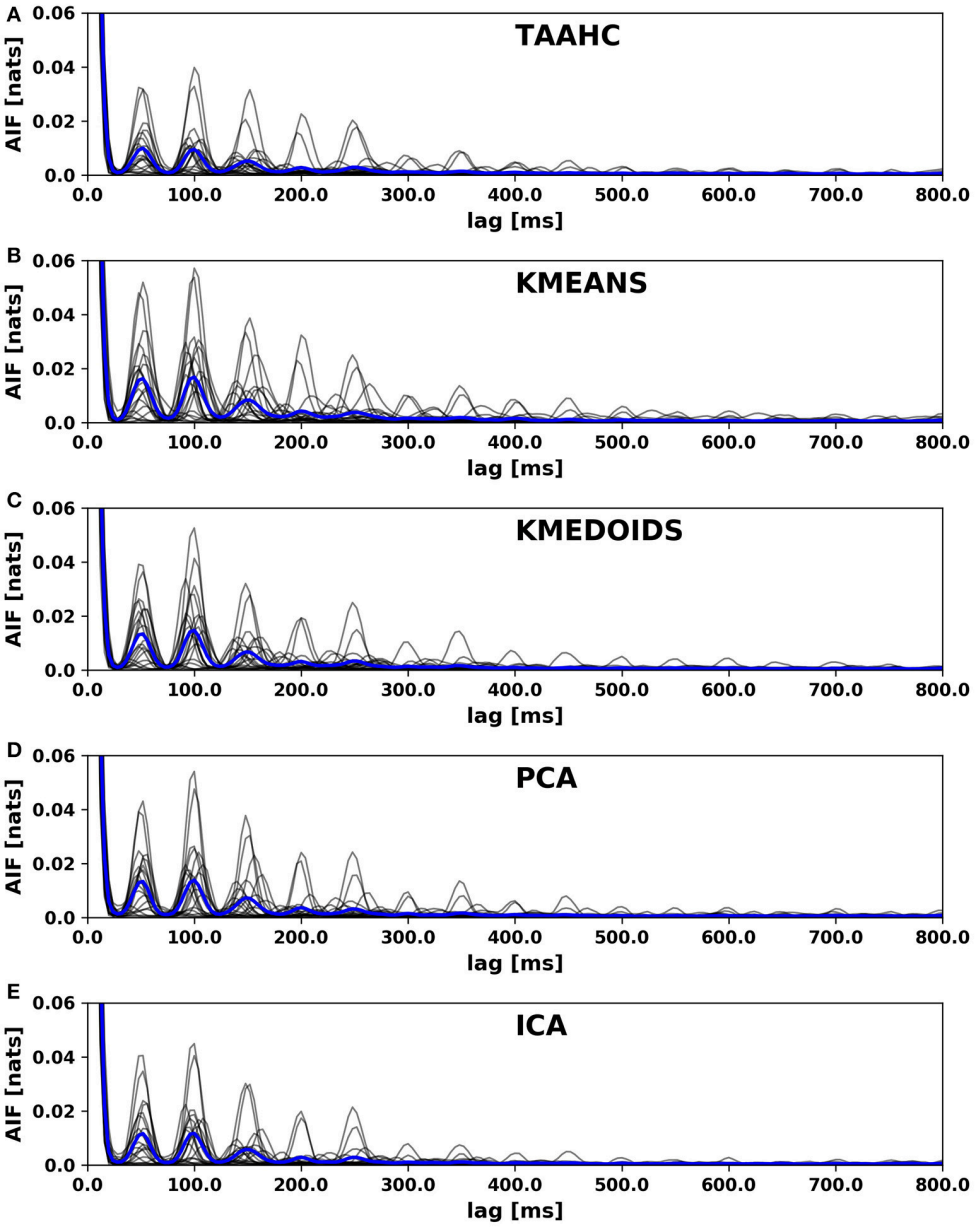
Autoinformation functions (AIF) of microstate sequences from different clustering algorithms. **(A)** Atomize and Agglomerate Hierarchical Clustering (AAHC), **(B)** Modified K-means algorithm, **(C)** Kmedoids clustering, **(D)** Principal Component Analysis (PCA), **(E)** Fast Independent Component Analysis (Fast-ICA). The individual AIFs for each subject are shown in light gray and the average AIF across all subject is shown in blue. The same periodicities are observed for all clustering algorithms.

The autoinformation functions of all subjects along with the arithmetic average of all AIFs is shown in Figure 2. We observe that all sequences reproduce periodicity with a main period of 50 ms. The main periodic component is twice the dominant (alpha) frequency of the underlying EEG data set. This effect, that we termed frequency doubling, is discussed in detail in von Wegner et al. (2017).

### 3.3. Correlations between static and dynamic properties

We will here summarize the mutual relations between static and dynamic properties for each clustering algorithm by means of cross-correlation matrices shown in the Supplementary Data section. The most consistent correlations found among all five clustering algorithms, that were statistically significant at a α = 0.05 level for at least four algorithms, were two correlation values related to the mixing time τ. First, τ was negatively correlated to $h_{n=8}^{\prime }$. Microstate sequences with larger τ values relax more slowly, or have a larger autocorrelation time. In other words, they fluctuate less and are more regular than ones with smaller τ values. This is in accordance with the interpretation of the entropy rate as an indicator of how much surprise the sequence produces per time step. Thus, the sequences with fast fluctuations have smaller τ values and larger entropy rates. Second, τ was positively correlated with the first AIF peak. This means, that microstate sequences with a longer characteristic periodicity also show slower relaxation times, as their whole dynamics evolve on a slightly larger time scale.

### 3.4. Information-theoretical quantities

#### 3.4.1. Markov property

We tested all microstate sequences for the Markov property of order 0, 1 or 2, and applied Bonferroni's multiple comparisons correction with respect to the 20 subjects tested. For all clustering algorithms and all subjects, the Markovian null hypothesis was rejected. It can therefore be concluded that all microstate sequences show memory effects extending beyond two time steps.

#### 3.4.2. Non-stationarity

Using K-means clustering, we recently reported non-stationary microstate transition matrices over time windows of 2–40 s, indicating that the transition probabilities between microstates can change during the time spans considered (von Wegner et al., 2017). We also found that for longer time windows, the proportion of non-stationary sequences decreases, suggesting a tendency toward stationary microstate transitions for time spans of approximately 20–40 s.

The results of stationarity testing for microstate sequences generated by the five clustering algorithms are shown in Table 4. For each algorithm (rows) and each block size *L* (columns), we show the proportion of subjects for which the null hypothesis of stationarity is rejected. A value of 0.85, for instance, means that 85% of the subjects (17/20) show non-stationary transition matrices, after Bonferroni correction of the 20 subjects studied. We observe the same general behavior, a large proportion of non-stationary segments for short blocks (*L* = 500 samples, or 2 s), and a tendency toward stationarity for longer blocks up to *L* = 10, 000 samples or 40 s. In total, ICA shows a significantly lower proportion of non-stationary data segments than the other algorithms, though following the same general tendency toward stationarity.

**Table 4:** Non-stationarity test for data blocks of size *L* (number of samples).

**Algorithm**	***L* = 500**	***L* = 1, 000**	***L* = 2, 500**	***L* = 5, 000**	***L* = 10, 000**
AAHC	0.85	0.85	0.75	0.70	0.45
K-means	0.95	0.90	0.75	0.65	0.40
K-medoids	0.95	0.95	0.90	0.65	0.55
PCA	0.80	0.70	0.55	0.55	0.40
ICA	0.60	0.40	0.25	0.30	0.20

#### 3.4.3. Periodic autoinformation function

The autoinformation functions for all subjects cized in Figure 2. For each algorithm, the individual AIF of each subject is plotted in light gray color and the mean AIF across all 20 subjects is shown as a bold blue line. We observe that all clustering algorithms show the same periodicities within the microstate sequences that we recently reported for maps computed from the modified K-means algorithm.

### 3.5. Hurst exponents of the microstate random walk

Even though the relation of Hurst exponents and long-range correlations is questionable in the case of non-stationary time series (von Wegner et al., 2016, 2018), *H* contains information about the multi-scale variance of the microstate time series and may therefore be used to characterize microstate sequences. We here present the Hurst exponent estimates computed by three different methods. Table 5 shows the average Hurst exponent estimates, all of which are larger than 0.5. Thus, all sequences show multi-scale variance effects and possibly long-range correlations, although the presence of non-stationarity would require further testing to detect these. The ANOVA test across the different clustering algorithms indicates significant differences between the clustering algorithms for DFA (*p* = 0.0055) and for DWT (*p* = 0.0006). For aggregated variance, no significant differences are found (*p* = 0.296).

**Table 5:** Mean Hurst exponent estimates.

**Algorithm**	**AV**	**DFA**	**DWT**
AAHC	0.586	0.634	0.589
K-means	0.586	0.656	0.593
K-medoids	0.575	0.649	0.590
PCA	0.591	0.660	0.584
ICA	0.567	0.630	0.589

*AV, aggregated variance; DFA, detrended fluctuation analysis; DWT, discrete wavelet transform*.

## 4. Discussion

In the present article, we compare statistical and information-theoretical properties of resting state EEG microstates derived from different clustering algorithms. We designed a systematic analysis of microstate properties that are independent of a specific map labeling in order to allow comparison of geometrically different microstate patterns and to analyze decompositions into more than the usual four microstate clusters. We consider static and dynamic properties of the microstate representation separately. Static properties involve similarity indices between microstate maps, the total percentage of data variance explained by the cluster representation, and the entropy of the microstate label distribution. Dynamic properties detect temporal dependencies in the microstate sequences, in analogy to the autocorrelation structure of metric time series.

We first observed that some algorithms can produce map geometries different from the four canonical maps described in the literature (Koenig et al., 2002) (Figure 1). However, inter-group correlations between microstate maps show large maximum correlation coefficients of 0.7–0.9 for most algorithm combinations. This result shows that there is at least one highly similar map combination found in both methods. The analysis of maximum intra-group correlations shows large similarities (ρ_max_ ≈ 0.8) between maps for AAHC, K-means and K-medoids, low intra-group correlations for ICA (ρ_max_ ≈ 0.4) and, by construction, zero correlations for PCA derived microstates. Further analyses of static properties show that the global explained variance (GEV) and the Shannon entropy of the computed sequences are within the same order of magnitude, but show statistically highly significant differences between algorithms.

The first two dynamic properties considered, i.e., the mixing time of the transition matrix and the entropy rate of the microstate sequences, also show significant differences between the algorithms, but *post-hoc* analysis suggests that for some combinations of clustering methods, these differences may be negligible. The location of the first AIF peak however does not show significant differences between the studied methods.

The information-theoretical tests for the Markov property of orders 0–2 show unequivocal results across the clustering methods, as Markovianity is rejected in all cases, even after Bonferroni correction. Thus, all microstate sequences seem to possess temporal correlations extending at least for 2 samples. Also the stationarity tests show a common behavior of non-stationary transitions for short time windows (e.g., 2 s) and a tendency toward stationarity for larger time windows (max. 40 s). The absolute proportion of non-stationary segments for ICA is lower than for the other algorithms, but follows the same tendency.

Finally, we assessed global temporal dependencies within microstate sequences with the information-theoretical autoinformation function (Figure 2). Our results show that the recently described phenomenon of periodically recurring microstates is invariant with respect to the clustering algorithm used (von Wegner et al., 2017).

It should be noted, however, that many of the quantities used here are mathematically well defined only for time-stationary time series. Yet, most EEG quantifiers, including such classical measures as the power spectral density, are not well defined in the presence of non-stationarity. However, these measures can be valid biomarkers if they reliably vary with the experimental condition studied. We focused on non-stationarity issues in three recent articles (von Wegner et al., 2016, 2017, 2018).

Our interpretation of these results is that the static properties contain a significant amount of information about the clustering algorithm used, e.g., the decorrelation property of PCA. Dynamic properties and information-theoretical quantities in particular, on the other hand, seem to reflect intrinsic properties of the underlying EEG signal that are independent of the algorithm employed to construct the microstate sequence. In total, we believe that our results convey a positive message with regard to the free choice of the clustering algorithm and to the use of information-theoretical methods for microstate research. Even though the algorithms may produce non-identical maps and diverging between-map correlations, dynamic properties directly related to the EEG and thus, to brain electrical activity, seem to be robust against the initial clustering algorithm. Further studies to explore the discussed properties in the context of cognitive task experiments or in ERP experiments are certainly needed. Our current results suggest that all clustering methods actually capture neurobiologically relevant properties of the EEG signal, rather than showing their own signature.

## Author contributions

FvW: designed the study, implemented the algorithms, performed data analysis and wrote the manuscript. PK: selected and pre-processed EEG data, performed data analysis and helped to produce tables and figures. HL: designed the study, selected data, analyzed data and wrote the manuscript.

## Conflict of interest statement

The authors declare that the research was conducted in the absence of any commercial or financial relationships that could be construed as a potential conflict of interest.
