# Supplementary material for: EEG Microstate Sequences From Different Clustering Algorithms Are Information-Theoretically Invariant
Source: Front Comput Neurosci. 2018 Aug 27;12:70. doi: 10.3389/fncom.2018.00070 (PMC6119811; doi:10.3389/fncom.2018.00070)
Supplement: Supplementary file 1 [file Data_Sheet_1.pdf]

Supplemental data for: EEG microstate sequences from different clustering algorithms are information-theoretically invariant

Frederic von Wegner, Paul Knaut, Helmut Laufs

August 14, 2018

We here give the full correlation matrices showing the correlation coefficients between static and dynamic microstate properties, for each algorithm. For each microstate algorithm, we obtain a set of $n = 20$ (number of subjects) static and dynamic values. Statistically significant values ($p < 0.05$) are indicated by a star ($*$).

Clustering algorithm: **AAHC**

	$\rho_{\max}$	GEV	h_{Sh}	τ	$h'_{n=8}$	AIF_1
$\rho_{\max}$	1.0	0.452^*	-0.397	-0.122	-0.133	0.138
GEV	0.452^*	1.0	-0.132	0.023	-0.338	0.304
h_{Sh}	-0.397	-0.132	1.0	-0.213	0.237	0.131
τ	-0.122	0.023	-0.213	1.0	-0.811^*	0.556^*
$h'_{n=8}$	-0.133	-0.338	0.237	-0.811^*	1.0	-0.593^*
AIF_1	0.138	0.304	0.131	0.556^*	-0.593^*	1.0

Clustering algorithm: **KMEANS**

	$\rho_{\max}$	GEV	h_{Sh}	τ	$h'_{n=8}$	AIF_1
$\rho_{\max}$	1.0	0.716^*	-0.143	-0.143	0.085	-0.127
GEV	0.716^*	1.0	-0.514^*	-0.044	-0.198	-0.234
h_{Sh}	-0.143	-0.514^*	1.0	0.220	0.130	-0.052
τ	-0.143	-0.044	0.220	1.0	-0.899^*	0.640^*
$h'_{n=8}$	0.085	-0.198	0.130	-0.899^*	1.0	-0.580^*
AIF_1	-0.127	-0.234	-0.052	0.640^*	-0.580^*	1.0

Clustering algorithm: **KMEDOIDS**

	$\rho_{\max}$	GEV	h_{Sh}	τ	$h'_{n=8}$	AIF_1
$\rho_{\max}$	1.0	−0.294	−0.107	0.384	−0.376	0.047
GEV	−0.294	1.0	−0.362	−0.444	−0.191	−0.108
h_{Sh}	−0.107	−0.362	1.0	0.132	0.578*	−0.219
τ	0.384	−0.444	0.132	1.0	−0.544*	0.662*
$h'_{n=8}$	−0.376	−0.191	0.578*	−0.543*	1.0	−0.626*
AIF_1	0.047	−0.108	−0.219	0.662*	−0.626*	1.0

Clustering algorithm: **PCA**

	$\rho_{\max}$	GEV	h_{Sh}	τ	$h'_{n=8}$	AIF_1
$\rho_{\max}$	1.0	0.062	0.076	0.149	−0.052	−0.233
GEV	0.062	1.0	−0.913*	−0.173	−0.738*	−0.083
h_{Sh}	0.076	−0.913*	1.0	0.282	0.684*	−0.032
τ	0.149	−0.173	0.282	1.0	−0.443	0.705*
$h'_{n=8}$	−0.052	−0.738*	0.684*	−0.443	1.0	−0.439
AIF_1	−0.233	−0.083	−0.032	0.705*	−0.439	1.0

Clustering algorithm: **ICA**

	$\rho_{\max}$	GEV	h_{Sh}	τ	$h'_{n=8}$	AIF_1
$\rho_{\max}$	1.0	−0.624*	0.233	0.256	−0.044	0.524*
GEV	−0.624*	1.0	−0.835*	−0.164	−0.601*	−0.312
h_{Sh}	0.233	−0.835*	1.0	0.183	0.748*	0.153
τ	0.256	−0.164	0.183	1.0	−0.482*	0.380
$h'_{n=8}$	−0.044	−0.601*	0.748*	−0.482*	1.0	−0.083
AIF_1	0.524*	−0.312	0.153	0.380	−0.083	1.0
